# Distinguishing benign and malignant thyroid nodules using plasma trimethylamine N-oxide, carnitine, choline and betaine

**DOI:** 10.1007/s00432-024-05666-w

**Published:** 2024-03-20

**Authors:** Liang Shi, Muhong Guo, Cuixiao Shi, Gu Gao, Xianghong Xu, Chuan Zhang, Jingjing Fu, Yudan Ni, Feng Wang, Xue Xue, Fei Yu

**Affiliations:** 1 Department of Nuclear Medicine, Nanjing First Hospital, Nanjing Medical University, Nanjing, 210006 China; 2 Department of General Surgery, Nanjing First Hospital, Nanjing Medical University, Nanjing, 210006 China; 3 Department of Health Management Center, Nanjing First Hospital, Nanjing Medical University, Nanjing, 210006 China; 4 Department of Endocrinology, Nanjing First Hospital, Nanjing, 210006 China

**Keywords:** Thyroid cancer, Trimethylamine N-oxide (TMAO), Carnitine, Choline, Betaine, Biomarker

## Abstract

**Purpose:**

Trimethylamine N-oxide (TMAO), a gut microbiome–derived metabolite, and its precursors (carnitine, choline, betaine) have not been fully examined in relation to thyroid cancer (TC) risk. The aim of this study was to assess the value of TMAO and its precursors in diagnosis of benign and malignant thyroid nodules.

**Methods:**

In this study, high-performance liquid chromatography-tandem mass spectrometry was utilized to measure the levels of plasma TMAO and its precursors (choline, carnitine, and betaine) in 215 TC patients, 63 benign thyroid nodules (BTN) patients and 148 healthy controls (HC). The distribution of levels of TMAO and its precursors among the three groups were compared by the Kruskal–Wallis test. Receiver operating characteristic curve (ROC) analysis was performed to evaluate the sensitivity, specificity, and the predictive accuracy of single and combined biomarkers.

**Results:**

In comparison to HC, TC showed higher levels of TMAO and lower levels of its precursors (carnitine, choline, and betaine) (all *P* < 0.001). Plasma choline (*P* < 0.01) and betaine (*P* < 0.05) were declined in BTN than HC. The levels of carnitine (*P* < 0.001) and choline (*P* < 0.05) were significantly higher in BTN than that in TC group. Plasma TMAO showed lower levels in TC with lymph node metastasis (101.5 (73.1–144.5) ng/ml) than those without lymph node metastasis (131 (84.8–201) ng/ml,* P* < 0.05). Combinations of these four metabolites achieved good performance in the differential diagnosis, with the area under the ROC curve of 0.703, 0.741, 0.793 when discriminating between TC and BTN, BTN and HC, TC and HC, respectively.

**Conclusion:**

Plasma TMAO, along with its precursors could serve as new biomarkers for the diagnosis of benign and malignant thyroid nodules.

## Introduction

Thyroid cancer (TC), a most common endocrine malignancy, has garnered significant attention due to rapidly increasing global incidence rate (Siegel et al. 2022). Papillary thyroid carcinoma (PTC) remains the most prevalent subtype, accounting for approximately 85% of all cases, followed by follicular, medullary, and anaplastic variants (Fagin and Wells 2016). Although the precise causes of TC remain elusive, extensive research has identified genetic predispositions, radiation exposure (head and neck region), environmental pollutants, high BMI, lifestyle and diet as potential contributors to the disease risk (Bogovic Crncic et al. 2020).

Ultrasonography (US) is the most important diagnostic tool and extremely sensitive for detecting thyroid nodules. Furthermore, US can be used to determine the size, number and characteristics of thyroid nodules, to guide fine-needle aspiration (FNA), and to detect lymph node metastasis (Gharib et al. 2016; Haugen et al. 2016). The US risk stratification system, known as the Thyroid Imaging Reporting and Data System (TIRADS), has been established to classify the malignancy risk of a thyroid nodule and subsequently recommend the requirement for US-guided FNA (Russ et al. 2017; Seo et al. 2015). Although US-guided FNA was a reliable and well accepted method for diagnosing TC (Seiberling et al. 2008), various complications such as bleeding, hoarseness, and infection can occur (Ha et al. 2018). Moreover, the accuracy of the FNA is influenced significantly by factors that are not solely related to the operator and cytologist, but also to the patient (e.g. size, location, homogeneity and vascularity of the nodule) (Blum 2000). Although the overall survival rate of TC is relatively high, early diagnosis is still of paramount importance (Liu et al. 2022b). Identifying effective early diagnostic TC biomarkers can minimize the need for unnecessary biopsies, prevent the misdiagnosis or undertreatment of a curable cancer, and to some extent relieve the patient’s anxiety (Sakorafas et al. 2010).

In order to meet the high bioenergetic and biosynthetic demands required for vigorous proliferation, cancer cells reprogram their metabolism, leading to the production of abnormal metabolic substances (Mayers and Vander Heiden 2015). Recent metabolomics studies have found that PTC is involved in metabolic abnormalities including glucose, lipid metabolism, amino acids and nucleotide metabolism (Farrokhi Yekta et al. 2018; Zhao et al. 2015). Trimethylamine N-oxide (TMAO) is a gut microbiome–derived metabolite, and is synthesized in multiple steps, with main precursor molecules being carnitine, choline, and betaine (Koeth et al. 2013; Wang et al. 2011). These precursor molecules have been shown to be present in abundance in red meat, eggs, milk, and certain fish products, including salmon (Zeisel and da Costa 2009). Over the past decade, high levels of TMAO have been linked to an increased risk of cardiovascular disease (Heianza et al. 2017), stroke (Tu and Xia 2024), diabetes (Dambrova et al. 2016), and chronic kidney disease (Tang et al. 2015). Furthermore, a growing number of studies have also identified a potential role for TMAO in tumor risk including colorectal cancer (Jalandra et al. 2021), breast cancer (Wang et al. 2022), prostate cancer (Mondul et al. 2015) and stomach cancer (Stonans et al. 2023). Potential mechanisms proposed for the carcinogenesis of TMAO may involve inflammation, oxidative stress, DNA damage, and protein misfolding (Jalandra et al. 2021).

There is evidence suggesting that higher levels of TMAO and its precursors in blood can be indicative of either a higher risk of malignancy or indeed its presence (Gatarek and Kaluzna-Czaplinska 2021). However, to date, no studies have specifically examined the relationship between TMAO and its precursors, as well as their potential impact on thyroid cancer risk. Blood-based biomarker tests may offer a cost-effective and non-invasive method for detecting or predicting the disease, but little is known about the levels of TMAO and its precursors in benign thyroid nodules (BTN) and TC. Herein we conducted a case-control study to reveal differences in the plasma TMAO and its precursors of TC, BTN, and healthy controls (HC) and discover non-invasive biomarkers for diagnosis of malignant and benign thyroid nodules.

## Material and methods

### Study population and sample collection

Plasma samples obtained from 215 patients diagnosed with TC, 63 patients diagnosed with BTN, and 148 HC were consecutively collected between November 2022 and November 2023 from the Nanjing First Hospital (Nanjing, China). The three subgroups were frequency matched on age and sex. Healthy controls were selected from health management center and they should have no history of cancer, thyroid related diseases, severe liver and kidney diseases, and have normal thyroid ultrasound. Patients with BTN were diagnosed based on ultrasound (nodules in American College of Radiology Thyroid Imaging Reporting and Data System (TI-RADS) category 3) or surgical histopathology. All TC were confirmed by surgical histopathology. TC patients were excluded if they had a history of any other cancers. Additionally, the patients’ tumor stage and lymph node stage were listed based on the Tumor/Node/Metastasis (TNM) classification. We obtained approval from the ethics committee of the Nanjing First Hospital and informed written consents from all participants were acquired.

3 ml of fasting peripheral blood was collected from all subjects and added to EDTA-K2 anticoagulant-containing tubes. After blood collection, the tubes were placed at room temperature (22–26 °C) and immediately centrifuged at 3000 rpm for 10 min, and the upper layer of plasma was collected and stored at − 80 °C until use.

### Plasma TMAO and its precursors analysis

Concentrations of plasma TMAO and its precursors (choline, carnitine, and betaine) were determined by high-performance liquid chromatography–tandem mass spectrometry (HPLC/MS–MS). We used trimethylamine-d9 N-Oxide TMAO (d9-TMAO, Cambridge Isotopes, Tewksbury, MA, USA) as an internal standard. Briefly, 50 ul of either the plasma or standards was mixed with 500 μl methanol containing 1 ug/ml internal standard and were subsequently centrifuged at 12000×*g* for 10 min to precipitate the proteins. Then, the remaining supernatant (5 μl) was injected into a Waters BEH C18 column (2.1 × 50 mm, 1.7 μm; Cat. No. 03433919615148, Massachusetts, USA) at a flow rate of 0.4 ml/min using an HPLC-pump, autosampler, and AB Sciex Triple Quad 4500 mass spectrometer (AB Sciex, FC, USA) with an electrospray ionization source. By mixing solvent A (0.1% formic acid in water) and solvent B (0.1% formic acid in methanol) in different ratios, starting at 10% B, a discontinuous gradient was generated to separate the analytes and then linearly increased to 80% B over 1.0 min, then hold for 1.8 min, then return to 10% B. Quantification of TMAO was performed using multiple reaction monitoring (MRM) transitions at m/z 76.1 → 59.0, d9‐TMAO at m/z 85.1 → 68.1, choline at m/z 104.1 → 60.1, carnitine at m/z 162.0 → 60.2, betaine at m/z 118.1 → 59.2.

### Statistical analysis

Shapiro–Wilk normality test was used to assess the normality of continuous variables’ distributions, and when *P* > 0.05, the distribution is considered to conform to the normal distribution. Continuous variables with normal distribution were described as mean ± standard deviation and compared by Analysis of Variance (ANOVA). Continuous variables with non-normal distribution were presented as median with an interquartile range, which were compared by the Kruskal–Wallis test. If a significant difference was found, a Dunn’s test was further applied to perform pairwise comparisons. Categorical variables were presented as counts and percentages, and tested using Pearson’s Chi-squared test. The correlation between levels of biomarkers was evaluated by Spearman correlation analysis. In addition, receiver operating characteristic curve (ROC) analysis was performed to evaluate the sensitivity, specificity, and the predictive accuracy of single and combined biomarkers. In this study, significance was defined as *P* < 0.05, and all statistical tests were two-tailed. Statistical analyses and graphing were performed by R software (version 3.6.1) and GraphPad Prism software.

## Results

### Clinical characteristics and TMAO and precursors expression in HC, BTN and TC groups

Overall, 426 participants including 148 HC, 63 BTN and 215 TC were collected for the analysis. All TC were confirmed by surgical histopathology and were classified as PTC. The distribution of sex and age in the three groups was consistent among all groups and was summarized in Table 1. In the TC group, the rate of lymph node metastasis was 38.14%. Among the TC cases, 96.28% were stage I and 3.72% were stage II.

**Table 1:** General information of all participants by subgroup

Variables	HC (n = 148)	BTN (n = 63)	TC (n = 215)	Statistic	*P*
Age	41.89 ± 11.26	47.57 ± 11.36	46.14 ± 10.68	55,681.82*	0.09*
Sex					
Male	63 (42.57%)	25 (39.68%)	76 (35.35%)		
Female	85 (57.43%)	38 (60.32%)	139 (64.65%)	1.97^#^	0.37^#^
Lymph node metastasis					
No	/	/	133 (61.86%)		
Yes	/	/	82 (38.14%)		/
TNM staging					
I	/	/	207 (96.28%)		
II	/	/	8 (3.72%)		/

*HC* healthy controls, *BTN* benign thyroid nodules, *TC* thyroid cancer

*Calculated from Analysis of Variance (ANOVA)

^#^Calculated from Pearson’s Chi-squared test

**Fig. 1:**
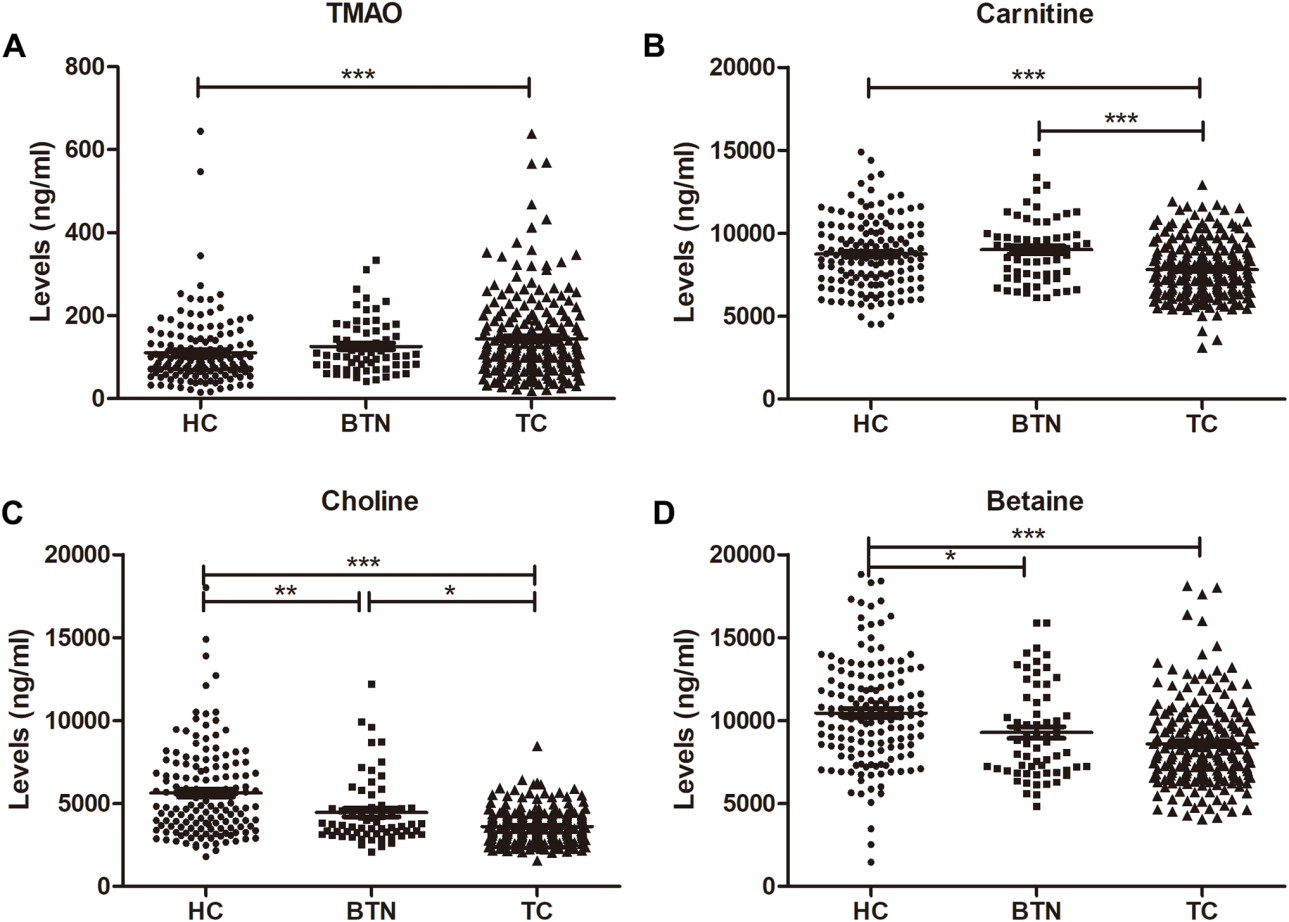
Plasma concentrations of TMAO, carnitine, choline and betaine in the HC group, BTN group and TC group. *HC* healthy controls, *BTN* benign thyroid nodules, *TC* thyroid cancer, *TMAO* microbiota-mediated trimethylamine N-oxide. *P < 0.05, **P < 0.01, ***P < 0.001

We examined the plasma TMAO and precursors (carnitine, choline, and betaine) expression among the HC group, BTN group and TC group (Table 2, Fig. 1). The results showed that the expression of TMAO in the TC group (median (interquartile range): 31.53 (26.87–37.77) ng/ml) was significantly higher than that in the HC group (118 (77–186.5) ng/ml, *P* < 0.001) (Fig. 1A). Plasma levels of carnitine were lower in the TC group (7630 (6510–8890) ng/ml) than that in the HC group (8665 (7227.5–10,100) ng/ml, *P* < 0.001) and BTN group (8970 (7525–9865) ng/ml,* P* < 0.001) (Fig. 1B). Concentrations of choline were recorded at 4860 (3585–7070) ng/ml in the HC group, 3690 (3195–4740) ng/ml in the BTN group and 3460 (2895–4160) ng/ml in the TC group (HC vs. BTN: *P* < 0.01; BTN vs. TC: *P* < 0.05; HC vs. TC: *P* < 0.001) (Fig. 1C). Plasma levels of betaine were declined in the TC group (8240 (6800–9895) ng/ml, *P* < 0.001) and BTN group (8720 (7070–11250) ng/ml,* P* < 0.05) when compared to the HC group (10,350 (8362.5–12,325) ng/ml) (Fig. 1D).

**Table 2:** Distribution of plasma concentrations of TMAO and precursors in subgroups

Variables	HC (n = 148)	BTN (n = 63)	TC (n = 215)	Statistic*	P*
TMAO (ng/ml)	91.5 (65–133)	104 (81.5–166)	118 (77–186.5)	14.59	6.78 × 10^–4^
Carnitine (ng/ml)	8665 (7227.5–10,100)	8970 (7525–9865)	7630 (6510–8890)	27.50	1.07 × 10^–6^
Choline (ng/ml)	4860 (3585–7070)	3690 (3195–4740)	3460 (2895–4160)	68.15	1.59 × 10^–15^
Betaine (ng/ml)	10,350 (8362.5–12,325)	8720 (7070–11250)	8240 (6800–9895)	38.04	5.49 × 10^–9^

*HC* healthy controls, *BTN* benign thyroid nodules, *TC* thyroid cancer, *TMAO* microbiota-mediated trimethylamine N-oxide

*Calculated from Kruskal–Wallis test

We further evaluated the value of plasma TMAO and precursors in stratifying TC to different lymph node metastasis status and clinical stages. From our results (Table 3), only plasma TMAO showed lower levels in TC with lymph node metastasis (101.5 (73.1–144.5) ng/ml) than those without lymph node metastasis (131 (84.8–201) ng/ml,* P* < 0.05). No other biomarkers exhibited statistically significant differences in lymph node metastasis and clinical staging of TC.

**Table 3:** Distribution of plasma concentrations of TMAO and precursors in different TC subgroups

Variables	Lymph node metastasis				TNM staging			
	No	Yes	Statistic*	P*	I	II	Statistic*	P*
TMAO (ng/ml)	131 (84.8–201)	101.5 (73.1–144.5)	5.48	**0.02**	119 (77–186.5)	111.5 (91.875–145.75)	0.02	0.88
Carnitine (ng/ml)	7670 (6520–8810)	7475 (6485–9320)	0.048	0.83	7650 (6520–8865)	7370 (5775–9347.5)	0.33	0.57
Choline (ng/ml)	3460 (2900–4130)	3430 (2882.5–4227.5)	0.15	0.70	3460 (2895–4160)	3290 (2895–3977.5)	0.005	0.94
Betaine (ng/ml)	8590 (7090–9940)	7915 (6597.5–9607.5)	1.79	0.18	8400 (6800–9970)	8040 (7522.5–8217.5)	1.05	0.31

*HC* healthy controls, *BTN* benign thyroid nodules, *TC* thyroid cancer, *TMAO* microbiota-mediated trimethylamine N-oxide

*Calculated from Kruskal–Wallis test

### Correlation between TMAO and precursors among different groups

Spearman correlation coefficient values of TMAO and precursors concentrations in the three groups (HC, BTN and TC) were calculated (Table 4). Weak to moderate positive correlation between choline and betaine levels was recorded in both females (R = 0.71, *P* < 0.001 in HC; R = 0.72, *P* < 0.001 in BTN, and R = 0.29, *P* < 0.001 in TC) and males (R = 0.72, *P* < 0.001 in HC; R = 0.55, *P* < 0.01 in BTN; R = 0.30, *P* < 0.01 in TC). Weak positive correlation was also observed between carnitine and betaine in females (R = 0.31, *P* < 0.01 in HC; R = 0.42, *P* < 0.01 in BTN, and R = 0.24, *P* < 0.01 in TC) and males (R = 0.34, *P* < 0.01 in HC; R = 0.26, *P* < 0.05 in TC). For carnitine and choline, weak positive correlation was only found in females with BTN (R = 0.49, *P* < 0.01) and with TC (R = 0.18, *P* < 0.05), and in healthy males (R = 0.36, *P* < 0.01) and males with TC (R = 0.26, *P* < 0.05). No significant or only very weak correlation was found between levels of TMAO and its precursors in the all subgroups.

**Table 4:**
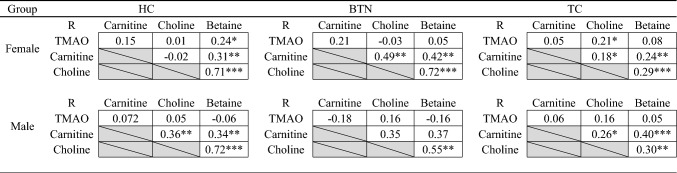
Correlation between TMAO and precursors among different groups

*HC* healthy controls, *BTN* benign thyroid nodules, *TC* thyroid cancer, *TMAO* microbiota-mediated trimethylamine N-oxide

*P < 0.05, **P < 0.01, ***P < 0.001

### The relationship between TMAO and precursors and the diagnosis of PTC

**Fig. 2:**
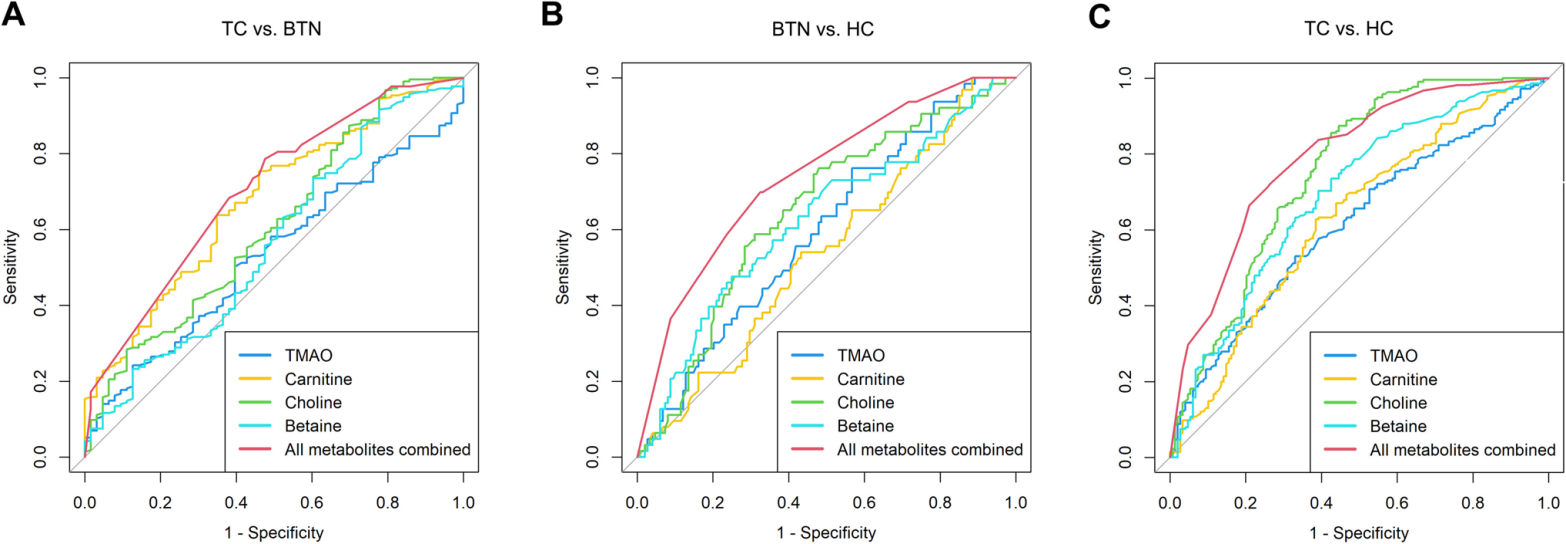
ROC curve analysis of the four single and combined metabolites. (**A**) TC vs. BTN, (**B**) BTN vs. HC, (**C**) TC vs. HC. *TC* thyroid cancer, *BTN* benign thyroid nodules, *HC* healthy controls, *TMAO* microbiota-mediated trimethylamine N-oxide

ROC curves were performed for the selected four metabolites. According to our results (Table 5 and Fig. 2), the AUCs of TMAO, carnitine, choline and betaine were 0.532, 0.676, 0.612 and 0.562 when discriminating between TC and BTN (Fig. 2A). Moreover, the AUCs of the four metabolites were 0.596, 0.538, 0.646 and 0.622 in differentiating BTN from HC (Fig. 2B). Furthermore, the AUCs of the four metabolites were 0.615, 0.631, 0.754 and 0.691 in the differential diagnosis of patients with TC and HC (Fig. 2C). Eventually, the multivariate logistic regression model was constructed based on the four metabolites. Combinations of these four metabolites achieved best performance in the differential diagnosis, with AUCs of 0.703, 0.741, 0.793 when discriminating between TC and BTN, BTN and HC, and TC and HC, respectively.

**Table 5:** Quantitative indexes of different models

Variables	TC vs. BTN				BTN vs. HC				TC vs. HC			
	Cut-off	AUC (95% CI)	Sensitivity (95% CI)	Specificity (95% CI)	Cut-off	AUC (95% CI)	Sensitivity (95% CI)	Specificity (95% CI)	Cut-off	AUC (95% CI)	Sensitivity (95% CI)	Specificity (95% CI)
TMAO (ng/ml)	190	0.532 (0.456–0.608)	24.2% (18.5–29.9%)	87.3% (79.1–95.5%)	81.45	0.596 (0.515–0.677)	76.2% (65.7–86.7%)	43.2% (35.3–51.2%)	112.5	0.615 (0.557–0.673)	53.0% (46.4–59.7%)	66.9% (59.3–74.5%)
Carnitine (ng/ml)	8915	0.676 (0.602–0.749)	54.0% (41.7–66.3%)	75.3% (69.6–81.1%)	6105	0.538 (0.455–0.622)	100.0% (100.0–100.0%)	11.5% (6.3–16.6%)	8175	0.631 (0.572–0.69)	61.5% (53.6–69.3%)	62.8% (56.3–69.3%)
Choline (ng/ml)	4670	0.612 (0.531–0.692)	30.2% (18.8–41.5%)	87.4% (83.0–91.9%)	4785	0.646 (0.566–0.726)	52.0% (44.0–60.1%)	76.2% (65.7–86.7%)	4550	0.754 (0.701–0.807)	57.4% (49.5–65.4%)	85.6% (80.9–90.3%)
Betaine (ng/ml)	11,050	0.562 (0.478–0.645)	27.0% (16.0–37.9%)	87.0% (82.5–91.5%)	8365	0.622 (0.538–0.706)	75.0% (68.0–82.0%)	47.6% (35.3–60.0%)	9550	0.691 (0.635–0.747)	60.8% (52.9–68.7%)	70.2% (64.1–76.3%)
Combined		0.703 (0.635–0.772)	78.6% (73.1–84.1%)	52.4% (40.0–64.7%)		0.741 (0.670–0.812)	69.8% (58.5–81.2%)	67.6% (60.0–75.1%)		0.793 (0.746–0.84)	72.1% (66.1–78.1%)	73.6% (66.6–80.7%)

*HC* healthy controls, *BTN* benign thyroid nodules, *TC* thyroid cancer, *TMAO* microbiota-mediated trimethylamine N-oxide, *AUC* area under curve, *CI* confidence interval

## Discussion

Metabolic abnormalities are a common occurrence in diseases, leading to the dysfunction of metabolic pathways and abnormal accumulations or deficiencies of metabolites. Metabolite biosignatures from human biofluids, bridging genotype, environment, and phenotype, offer compelling biomarker candidates for clinical diagnosis, classification and prognosis prediction (Qiu et al. 2023). The identification of differential metabolites or metabolic pathway alterations can provide valuable insights into the pathophysiology of diseases, improve the precision and accuracy of patient diagnosis and risk prediction, and aid in the discovery of potential therapeutic targets (Alexander et al. 2023; Liu et al. 2022a; Perea-Gil et al. 2022). The absence of early biomarkers in TC may lead to inadequate diagnosis and unfavorable outcomes. Therefore, it is crucial to develop noninvasive diagnosis and monitoring methods that exhibit high levels of specificity and accuracy.

To the best of our knowledge, this is the first targeted study to investigate the association between plasma TMAO levels and TC risk in humans. We observed a significant increase in plasma TMAO levels in TC patients compared to HC, but no significant difference was observed when compared to BTN patients. In this study, alterations in blood TMAO levels were observed that were in accordance with a recent study conducted by Chinese researchers, who performed untargeted serum metabolomic profiling in 30 TC and 27 HC and found TMAO presented significant higher level in the TC (Zhou et al. 2017). TMAO is a compound produced by the gut microbiota from dietary nutrients. It has been increasingly recognized as a potential risk factor for various diseases, including cancer (Oellgaard et al. 2017). A meta-analysis has examined the associations between TMAO concentrations and cancer risk, and has demonstrated positive correlations between high TMAO levels and increased risks of colorectal cancer, prostate cancer, primary liver cancer, and pancreatic cancer (Li et al. 2022). Our study offers insights into the potential role of TMAO in TC, suggesting that it may serve as a biomarker for TC development. In our study, no significant or only very weak correlation was observed between TMAO and its precursors. Similarly, Zhou et al. supported our findings by demonstrating that there is no significant correlation between carnitine and TMAO (Zhou et al. 2017). These results suggest that the relationship between these compounds may not be as straightforward as previously thought, calling for further investigation into their interactions and potential impact on health.

Furthermore, we observed that only plasma TMAO, rather than its precursor, can distinguish TC patients with different lymph node metastasis statuses. Interestingly, TMAO levels were found to be lower in the lymph node metastasis group compared to the non-metastasis group. In alignment with our study, Seo et al. conducted research to explore the potential role of targeted metabolites, including choline, in predicting lymph node metastasis in patients with PTC. They discovered that none of the metabolites tested could effectively discriminate the presence of lymph node metastasis (Seo et al. 2018). Overall, TMAO can induce tumor growth by promoting cell proliferation, angiogenesis, inflammation or oxidative stress (Chan et al. 2019; Yang et al. 2022). However, recent work strongly supports the idea that TMAO can drive immune activation and promotes antitumor immunity (Mirji et al. 2022; Wang et al. 2022). The observed contrasting roles of TMAO in TC development and tumor progression may be attributed to the complex interactions between TMAO and the tumor microenvironment. In the initial stages of TC development, TMAO promotes tumor growth by modulating cell proliferation, invasion, and angiogenesis. However, as the disease progresses, tumors reduce TMAO levels through specific mechanisms, potentially to evade immune surveillance and facilitate metastasis. This dual effect highlights the complex and dynamic nature of TMAO in tumorigenesis and progression, and underscores the need for further research to fully understand its role in cancer biology.

According to the literatures, choline is related to TC, but the results are controversial. Ryoo et al. utilized nuclear magnetic resonance (NMR) spectroscopy to analyze the metabolome of percutaneous FNA specimens and they discovered that choline levels were elevated in PTC tissue compared to benign nodules (Ryoo et al. 2016). Deja et al. applied NMR-based metabolic profiling and found choline was higher in TC tissues compared to healthy thyroid tissue (Deja et al. 2013). While some studies reported that choline was decreased in TC tissue than benign nodules (Miccoli et al. 2012; Torregrossa et al. 2012). In addition to tissue samples, experts also conduct metabolic profiling research using peripheral blood samples. Consistent with our research, Zhao et al. revealed a decrease in serum choline levels in PTC patients compared to healthy controls (Zhao et al. 2015). However, Yekta et al. discovered that serum choline levels were higher in PTC patients than in healthy controls (Farrokhi Yekta et al. 2018). Carnitine is a derivative of amino acids and an essential nutrient involved in the metabolism of lipids in mammals and our result of low carnitine levels in the serum of TC patients is inconsistent with a previous study (Zhou et al. 2017). Nevertheless, higher blood levels of L-carnitine were demonstrated in male gastric cancer (Stonans et al. 2023). To date, no studies have reported changes of betaine levels in TC. Our study first demonstrated that plasma betaine levels were elevated in TC patients than BTN patients and HC. A recent meta-analysis of cohort studies has provided further support for our findings, indicating that higher levels of betaine may indeed reduce the risk of cancer incidence (Youn et al. 2019).

We believe that the credibility of our results is enhanced because we utilize targeted metabolic techniques and have a larger sample size compared to previous studies. In our study, we observed an overall increasing trend in TMAO levels and a corresponding decreased trend in the levels of its precursors across the three groups of HC, BTN, and TC. This phenomenon may be partly explained by the following possible reason: compared to healthy individuals, the abundance and diversity of intestinal microflora may exhibit significant changes (e.g. increased number and diversity of *Clostridium*) during the development of benign and malignant nodules, facilitating the conversion of precursors to TMAO (Backhed 2013; Koeth et al. 2013). As our study did not include data on gut microbiota, further research incorporating microbial analysis is warranted to validate our hypotheses. However, there are some limitations in this study. First, as a retrospective case-control design with relatively small sample size, we cannot conclude definitively that there is an association between plasma TMAO and its precursors concentration and TC. Second, dietary information that could influence plasma TMAO levels was not gathered (Hamaya et al. 2020). In the future, larger prospective studies with more comprehensive designs are needed to further investigate the relationship between TMAO and its precursors levels and TC risk. Further studies are also needed to fully understand the mechanisms underlying the effects of TMAO and its precursors on TC and to determine whether it can serve as a potential target for cancer prevention and treatment.

In conclusion, we found that plasma TMAO was elevated in TC patients, whereas its precursors (carnitine, choline and betaine) were decreased. TMAO and its precursors may serve as potential biomarkers for discriminating benign from malignant thyroid nodules.

## Data Availability

The datasets generated and/or analysed during the current study are available from the corresponding author on reasonable request.
